# Fungal Foe and Mechanical Hearts: A Retrospective Case Series on *Candida auris* Bloodstream Infection With Left Ventricular Assist Devices

**DOI:** 10.1093/ofid/ofae286

**Published:** 2024-05-17

**Authors:** Ajay Kerai, Saumil Doshi, Ashley Laleker, Anjali Majumdar

**Affiliations:** Department of Internal Medicine and Department of Infectious Disease, MedStar Washington Hospital Center, Washington, District of Columbia, USA; Department of Internal Medicine and Department of Infectious Disease, MedStar Washington Hospital Center, Washington, District of Columbia, USA; Department of Internal Medicine and Department of Infectious Disease, MedStar Washington Hospital Center, Washington, District of Columbia, USA; Division of Allergy and Infectious Disease, Department of Medicine, Rutgers-Robert Wood Johnson Medical School, New Brunswick, New Jersey, USA

**Keywords:** antifungals, *Candida auris*, candidemia, heart transplant, left ventricular assist device

## Abstract

No guidelines currently exist for the management of *Candida auris* bloodstream infection in patients with left ventricular assist devices (LVADs). We aim to share our management experience through this retrospective case series outlining 15 episodes of *C auris* candidemia identified in 7 patients over 18 months. The initial source of candidemia was central venous catheter in 5 patients, driveline exit site infection in 1 patient, and possible pump infection in 1 patient. All patients were initially treated with micafungin. Despite susceptibility to micafungin, 4 patients experienced recurrent *C auris* candidemia. All patients died within 1 year of their first episode of *C auris* candidemia. Source control is challenging in patients with LVADs, and strict infection prevention measures should be practiced. More studies are needed to evaluate the role of newer antifungal agents, use of combination antifungal regimens, and impact on morbidity in patients with LVADs.

## BACKGROUND

Because of transplant candidacy barriers and limited donors, left ventricular assist devices (LVADs) are increasingly used as bridge to transplantation or destination therapy in advanced heart failure. Infection is a common LVAD complication, accounting for 5%–6% of LVAD-related deaths [1, 2]. Approximately 12%–60% of patients with LVADs develop bloodstream infections (BSIs), categorized as ventricular assist device (VAD)-related infections by the 2011 working definitions from the International Society for Heart and Lung Transplantation (ISHLT) [3–5]. These BSIs are commonly the result of bacteria such as *Staphylococcus aureus*, *Staphylococcus epidermidis, Pseudomonas aeruginosa*, and other gram-negative bacilli [4, 5]. In contrast, fungal VAD-related BSIs are less common (6%–8%) and usually to the result of *Candida parapsilosis*, *Candida glabrata*, and *Candida albicans* [4, 5]. Fungal BSIs are less likely to achieve clinical cure compared to bacterial infections (17% vs 56%) and have higher all-cause mortality (91% vs 62%) [5, 6].

There is no specific literature on *Candida auris* BSI management in patients with LVADs. *C auris* is a multidrug-resistant (MDR) yeast causing infections in patients with healthcare exposure with associated mortality up to 70% [7, 8]. *C auris* incidence is increasing at an alarming rate, with 3851 clinical cases reported in the USA from 2021 to 2022 [9, 10]. The World Health Organization has categorized *C auris* into the critical priority group [11].

Patients with LVADs have frequent healthcare exposure and may also have central venous catheters (CVCs), further increasing *C auris* infection risk [12]. The Infectious Diseases Society of America candidiasis guidelines recommend treating candida-related LVAD infections similarly to native valve endocarditis, followed by fluconazole chronic suppression if possible [13]. However, 90% of *C auris* isolates in the USA are fluconazole-resistant [14]. The Centers for Disease Control and Prevention (CDC) offer treatment guidelines for *C auris* infections, but no guidance exists for LVAD-related infections [15].

Through this retrospective case series of 7 patients with LVAD-associated *C auris* BSIs, we aim to share our experience of infection management and patient outcomes.

## METHODS

MedStar Health Research Institute institutional review board approval was obtained. We included nonpregnant patients ≥18 years old with LVAD who had ≥1 blood culture(s) growing *C auris* and admitted at a tertiary care hospital from 1 January 2021 through 30 June 2022. Through chart review, we extracted data including demographics, comorbidities, healthcare or nursing facility exposure 90 days before candidemia, antifungal treatment, antifungal minimal inhibitory concentrations, concomitant infections, and candidemia source. Initial candidemia episode was defined as the first blood culture that grew *C auris.* Subsequent candidemia episodes, or recurrences, were defined as a blood culture growing *C auris* after a negative blood culture in a patient with a prior *C auris* candidemia episode. Candidemia episodes were categorized according to 2011 ISHLT VAD infection definitions [3]. We reviewed outcomes such as time to clear candidemia, number of recurrences, and death, adjudicated by S. D. and A. M.

Our local health department recommended targeted *C auris* screening starting in April 2021 [16]. Patients admitted from high-risk facilities were screened for *C auris* colonization via axillary and groin swab culture. All patients with a positive *C auris* screen or culture were placed on contact isolation for index and future hospitalizations.

## RESULTS

Baseline characteristics are outlined in Table 1. Seven patients with 15 episodes of *C auris* BSI were identified. Four patients had at least 1 candidemia recurrence. All patients had prolonged (>15 days) exposure to a healthcare or nursing facility within 90 days of initial candidemia. Initial candidemia sources were identified as: CVC (5 patients), possible pump infection (1 patient), and driveline exit site infection (DLES) (1 patient). All patients received micafungin as initial treatment. Time from LVAD implant to first candidemia episode ranged from 112 to 2374 days and time from initial *C auris* BSI to death ranged from 12 to 358 days. Patient 1 was admitted before *C aris* screening initiation in April 2021, patient 2 had a positive screen 2 months before admission, and the remaining patients did not meet screening criteria.

**Table 1.** Patient Characteristics

Characteristic	All Patients (n = 7)
Age, y, mean (range)	55 (35–73)
Sex	
Male	5 (71)
Female	2 (29)
Race or ethnic group	
African American	7 (100)
LVAD	
Destination therapy	6 (86)
Bridge-to-transplant	1 (14)
LVAD type	
HeartMate 3	6 (86)
HeartWare	1 (14)
Comorbidities	
Chronic respiratory failure, tracheostomy status	5 (71)
Diabetes mellitus	4 (57)
End-stage renal disease	4 (57)
Obesity	4 (57)
Concurrent bacterial infections	6 (86)
Time from LVAD implant to initial episode of candidemia	
Within year 1	4 (57)
From years 2 to 5	2 (29)
After 5 y	1 (14)
Days to clear each episode of candidemia, mean (range)	21 (2–132)
Length of hospitalization, d, mean (range)	33 (18–235)

Data are presented as no. (%) unless otherwise stated.

Abbreviations: AICD, automatic implantable cardioverter defibrillator; LVAD, left ventricular assist device.

## BRIEF CASE SUMMARIES

Cases are summarized in Table 2.

**Table 2.** Summary of Cases

	Patient 1	Patient 2	Patient 3	Patient 4	Patient 5	Patient 6	Patient 7
Age (y), gender, race/ethnicity	73, male, AA	62, male, AA	52, male, AA	39, female, AA	35, male, AA	65, female, AA	61, male, AA
LVAD type (goal)	HeartMate 3 (DT)	HeartMate 3 (DT)	HeartMate 3 (DT)	HeartMate 3 (BTD)	HeartMate 3 (DT)	HeartWare (DT)	HeartMate 3 (DT)
Infection(s) before initial *C auris* candidemia	*Mycobacterium abscessus* pump infection	VRE *Enterococcus faecium* mediastinitis	MRSA mediastinitis, MDR *Pseudomonas aeruginosa* deep driveline infection	*Candida parapsilosis* candidemia	VRE *Enterococcus faecium* AICD IE, MDR *P aeruginosa* DLES infection	MSSA and *Pseudomonas aeruginosa* DLES infection, MDR *Acinetobacter baumannii* bacteremia	ESBL *Klebsiella pneumoniae* DLES infection and pneumonia, CRE *Klebsiella pneumoniae* bacteremia
Concurrent bacterial infections at time of candidemia	MDR *P aeruginosa* pneumonia	MDR *A baumannii* pneumonia, CRE *K pneumoniae* pneumonia, VRE *E faecium* and MRSA bacteremia	MDR *P aeruginosa* and *E coli* DLES infection, MDR *P aeruginosa* bacteremia	*S epidermidis* bacteremia, Polymicrobial pneumonia *(ESBL E coli,* CRE *K pneumoniae,* *S maltophilia)*	MDR *P aeruginosa* PNA	Polymicrobial deep driveline infection (MDR *A baumannii*, *P aeruginosa*, and MRSA), MRSA bacteremia	None
AICD present	No	Yes	Yes	Yes	Yes	Yes	Yes
TTE for IE	Not done	No vegetation	Not done	No vegetation	Not done	No vegetation	No vegetation
TEE for IE	Not done	Not done	AICD lead vegetation, AICD explanted (episode 3.2)	Not done	AICD lead vegetation, AICD explanted prior to candidemia	AICD lead vegetation, AICD not explanted	Not done
Time from LVAD implant to initial candidemia	201 d	185 d	254 d	112 d	889 d	1846 d	473 d
Time from initial candidemia to death	69 d	50 d	266 d	358 d	12 d	156 d	70 d
Candidemia present at time of death	No	No	Yes	No	No	No	No
Episodes of candidemia	1	2	3	5	1	2	1
Source(s) implicated for *C auris* candidemia (2011 ISHLT definitions) [3, 18]	Episode 1.1: CVC (LVAD-related)	Episode 2.1: CVC (LVAD-related) Episode 2.2: CVC (LVAD-related)	Episode 3.1:PICC (LVAD-related) Episode 3.2: IE (LVAD-related) Episode 3.3: Possible Pump (LVAD-specific)	Episodes 4.1–4.5: Possible Pump (LVAD-specific)	Episode 5.1: CVC (LVAD-related)	Episode 6.1: DL (LVAD-specific) Episode 6.2: IE: (LVAD-related)	Episode 7.1: CVC (LVAD-related)
Antifungal therapy	Episode 1.1: MICA 150 mg daily, 6 wk	Episode 2.1: MICA 100 mg daily, 6 wk Episode 2.2: MICA 100 mg daily, 6 wk	Episode 3.1: MICA 100 mg daily, 2 wk Episode 3.2: MICA 150 mg daily + AMB 500 mg daily + 5FC 2 g every 6 h. Once cleared: CASP 150 mg daily for 6 wk then POS 300 mg daily suppression Episode 3.3: MICA 150 mg daily + 5FC 1.75 g every 6 h indefinitely	Episode 4.1: MICA 150 mg daily continued into episode 4.2 Episodes 4.2–4.5: MICA 150 mg daily + AMB 5 mg/kg/daily indefinitely	Episode 5.1: MICA 150 mg daily, 12 d	Episode 6.1: MICA 150 mg daily, 6 wk Episode 6.2: MICA 150 mg daily + POSA 300 mg daily, 12 wks then POSA 300 mg daily suppression	Episode 7.1: MICA 150 mg daily, 4 wk
Days to clear each candidemia episode	Episode 1.1: 2 d	Episode 2.1: 3 d Episode 2.2: 7 d	Episode 3.1: 4 d Episode 3.2: 70 D Episode 3.3: Persistent	Episode 4.1: 6 d Episode 4.2: 22 d Episode 4.3: 8 d Episode 4.4: 8 d Episode 4.5: unknown^a^	Episode 5.1: 8 d	Episode 6.1: 7 d Episode 6.2: 11 d	Episode 7.1: 5 d

Abbreviations: 5FC, flucytosine; AA, African American; AICD, automatic implantable cardioverter defibrillator; AMB, liposomal amphotericin B; BTD, Bridge to Decision; CASP, caspofungin; CRE, carbapenem-resistant Enterobacterales; CVC, central venous catheter; DL, driveline; DLES, driveline exit site; DT, destination therapy; ESBL, extended-spectrum beta-lactamase; IE, infective endocarditis; LVAD, left ventricular assist device; MDR, multidrug resistant; MICA, micafungin; MRSA, methicillin-resistant *Staphylococcus aureus*; MSSA, methicillin-sensitive *Staphylococcus aureus*; PICC, peripherally inserted central catheter; POSA, posaconazole; TEE, transesophageal echocardiogram; TTE, transthoracic echocardiogram; VRE, vancomycin-resistant enterococci.

^a^Patient was discharged after episode 4.5 on lifelong antifungal therapy and readmitted 141 d later. No culture data were available to document candidemia clearance between admissions.

Case 1: A 73-year-old male with end-stage renal disease on hemodialysis via CVC on intravenous antibiotics for presumed LVAD pump infection was admitted from nursing home with septic shock from *C auris* and *C albicans* candidemia and MDR *P aeruginosa* pneumonia. The CVC was removed, micafungin was started, and blood cultures cleared after 2 days. He was discharged on micafungin for 6 weeks and antibacterials but readmitted 25 days later with MDR *P aeruginosa* pneumonia. He was transitioned to comfort care and died.

Case 2: A 62-year-old male with end-stage renal disease on hemodialysis via tunneled CVC, LVAD-related vancomycin-resistant *Enterococcus faecium* mediastinitis, presented from nursing home with septic shock from *C auris* candidemia and bacterial pneumonia. Micafungin was started, CVC was removed, blood cultures cleared after 3 days, and he was discharged on 6 weeks of micafungin and antibacterials. He was readmitted 17 days later with septic shock; blood cultures grew methicillin-resistant *S aureus* (MRSA), *Enterococcus faecium*, and *C auris*. Micafungin was continued with BSI clearance after 7 days. Transesophageal echocardiogram (TEE) was not done because of hemodynamic instability, and the patient died after transition to comfort care.

Case 3: A 52-year-old male on antibacterial suppression for prior LVAD infection presented with DLES discharge and *C auris* BSI. Micafungin was started and BSI cleared in 4 days. He underwent DLES debridement; tissue cultures grew MDR *P aeruginosa*. He was discharged on micafungin for 2 weeks and antibacterials. He was readmitted 80 days later with DLES discharge and recurrence of *C auris* BSI. DLES debridement tissue cultures grew *P aeruginosa* and *C auris.* He was started on micafungin and liposomal amphotericin B (AMB). TEE revealed automatic implantable cardioverter-defibrillator (AICD) lead vegetation resulting in AICD extraction. AICD culture grew *C auris* and 70 days later candidemia cleared. He was discharged on caspofungin, oral posaconazole, and antibacterials. He was readmitted 9 days later with *C auris* candidemia and MDR *P aeruginosa* bacteremia. He was started on micafungin, flucytosine, and antibiotics. The patient had persistent candidemia, was transitioned to comfort care, and died.

Case 4: A 39-year-old female underwent LVAD implantation complicated by cardiac arrest requiring percutaneous gastrostomy tube placement complicated by peritonitis requiring exploratory laparotomy. She developed *C auris* candidemia 125 days into hospitalization, was started on micafungin, and BSI cleared in 6 days. Candidemia recurred after 8 days and persisted; AMB was added. Transthoracic echocardiogram did not reveal vegetations and TEE was not pursued as the source was attributed as possible pump infection. She was discharged on micafungin and AMB for presumed pump infection. She was readmitted 69 days later with *C auris* and *Candida krusei* BSI with *Stenotrophomonas* pneumonia. Blood cultures cleared after 8 days and she was discharged on lifelong micafungin and AMB. Nine days later, she was readmitted with a *C auris* BSI and again discharged on micafungin and AMB. No culture data were available to document clearance until readmission 141 days later for shock and right ventricular failure. She subsequently passed away.

Case 5: A 35-year-old male with LVAD, history of *C glabrata* peritonitis, vancomycin-resistant *Enterococcus* AICD endocarditis on antibacterial suppression, presented with sepsis. DLES discharge culture grew *P aeruginosa*. DLES was not debrided because of hemodynamic instability. TEE revealed lead vegetation and AICD was explanted. On hospitalization day 60, he developed *C auris* BSI and MDR *P aeruginosa* pneumonia. He was started on micafungin and antibiotics. Candidemia cleared in 8 days; however, he developed worsening lung abscess and eventually died.

Case 6: A 65-year-old female with history of methicillin-sensitive *Staphylococcus aureus* pump infection on chronic antibiotic suppression presented with DLES discharge. Her long-term peripherally inserted central catheter was removed and she underwent DLES debridement. Admission blood cultures grew MRSA and *C auris*, whereas DLES debridement culture grew MRSA, *C auris*, MDR *P aeruginosa*, and MDR *Acinetobacter baumannii*. Candidemia cleared after 7 days of micafungin and she was discharged on micafungin and antibacterials. She was readmitted 38 days later with AICD shocks; blood cultures grew *C auris.* She was started on micafungin and AMB with tunneled CVC removal. TEE revealed AICD lead vegetation, but she was deemed high risk for explant. Candidemia cleared within 11 days and she was discharged on antibacterials, micafungin, and posaconazole. She was readmitted 34 days later with sepsis, respiratory failure, and no laboratory evidence of candidemia. The patient desired comfort care and died.

Case 7: A 61-year-old male with prior DLES infection presented with DLES discharge. Hospitalization was complicated by cardiac arrest from septic shock and bowel ischemia requiring resection. One month of empiric antibiotics and micafungin were completed. Total parenteral nutrition was started via tunneled CVC. He developed *C auris* candidemia, which prompted CVC removal. Micafungin was started, candidemia cleared in 5 days, and the patient was discharged. He died 51 days later from gastrointestinal bleeding. No culture data were available from the time of discharge to readmission.

## ANTIFUNGAL SUSCEPTIBILITY DATA

All isolates were susceptible to micafungin per CDC breakpoints and 13/15 were resistant to fluconazole (Table 3). Despite susceptibility to micafungin, 4 patients had candidemia recurrences.

**Table 3.** Antifungal Susceptibility Profile of Candidemia Episodes

Antifungal	ANID	CASP	MICA	FLUC	ITRA	POSA	VORI	AMB	5FC
CDC MIC breakpoints (µg/mL)	**≥ 4**	**≥ 2**	**≥ 4**	**≥ 32**	**No data^a^**	**No data^a^**	**No data^a^**	**≥ 2**	**No data^a^**
Episode 1.1	≤ 0.016	0.12	0.016	0.25	0.03	0.016	≤ 0.008	0.25	16
Episode 2.1	0.12	0.12	0.12	≥ 512	0.25	0.12	2	1	0.12
Episode 2.2	0.5	≥ 16	0.5	≥ 512	0.25	0.25	2	2	0.12
Episode 3.1	0.25	≥ 16	0.25	≥ 512	0.25	0.12	1	1	0.12
Episode 3.2	0.25	0.25	0.12	128	0.12	0.06	0.5	1	0.12
Episode 3.3	0.25	0.25	0.25	≥ 512	≥ 32	≥ 16	≥ 16	2	0.12
Episode 4.1	0.25	0.5	0.12	≥ 512	0.25	0.12	2	1	0.12
Episode 4.2	0.25	0.25	0.12	≥ 512	0.25	0.12	4	1	0.12
Episode 4.4^b^	0.12	0.06	0.06	128	0.12	0.03	0.5	4	0.12
Episode 4.5	0.12	0.12	0.06	128	0.12	0.03	0.25	4	0.12
Episode 5.1	0.25	0.25	0.25	≥ 512	0.25	0.12	2	1	0.12
Episode 6.1	0.12	0.06	0.12	≥ 512	1	0.25	8	2	0.12
Episode 6.2	0.5	≥ 16	1	≥ 512	0.5	0.25	8	4	0.12
Episode 7.1	0.25	0.12	0.25	256	0.25	0.06	1	2	≤ 0.06
Number of resistant episodes	0	3	0	13	…	…	…	7	…

Abbreviations: 5FC, flucytosine; AMB, liposomal amphotericin B; ANID, anidulafungin; CASP, caspofungin; CDC, Centers for Disease Control and Prevention; FLUC, fluconazole; ITRA, itraconazole; MIC, minimal inhibitory concentration; MICA, micafungin; POSA, posaconazole; VORI, voriconazole.

^a^CDC offers guidance to consider fluconazole susceptibility as surrogate for second-generation triazole susceptibility.

^b^Episode 4.3 did not undergo susceptibility testing.

## DISCUSSION

Currently, no specific guidelines exist on the management of *C auris* BSI in patients with LVADs. We report a novel case series of 7 cases with 15 episodes of *C auris* BSI in LVAD patients, including management and outcomes. *C auris* BSIs were difficult to eradicate in the setting of LVADs. Six patients had concomitant MDR infections and all died within 1 year of initial *C auris* BSI. This suggests that *C auris* BSI is a poor prognostic indicator in patients with LVADs and may require aggressive upfront therapy.

Source control was challenging in non–CVC-related infections. Though pump exchange or heart transplantation can be pursued for refractory pump infections, cases 3 and 4 were deemed high-risk candidates [17, 18, 19]. Infectious Diseases Society of America guidelines also recommend AICD removal with candidal cardiac infections along with chronic antifungal treatment for LVADs that cannot be removed; case 6 was deemed high risk for AICD explantation [13].

Despite susceptibility to micafungin, 4 patients had recurrent BSI. This reflects the ability of *C auris* to make biofilms and the associated challenges of achieving adequate source control [12]. Four episodes were treated with combination antifungal therapy; there is minimal guidance regarding this clinical practice [20]. Newer antifungal agents such as fosmanogepix, ibrexafungerp, and opelconazole have demonstrated activity against *C auris* biofilm formation and may become future options to treat *C auris* infections in LVAD patients [21].

Although no formal investigation was conducted, the cases were unlikely to be secondary to a single center–associated outbreak. All patients had exposure to different healthcare facilities before candidemia, 6 candidemia episodes were present on admission, 1 episode occurred in a patient with prior positive *C auris* screen, and initial episodes were spread over time (13 months). Once identified, all patients were placed on contact isolation and routine and terminal cleaning was performed per CDC recommendations.

The descriptive nature of a case series and small sample size limits assessment of *C auris* attributable mortality. All patients died within 1 year of their first episode of *C auris* candidemia. One patient was candidemic, whereas 6 patients had MDR bacterial infections at time of death, making it difficult to establish a causal relationship between *C auris* BSI and mortality. Most LVADs were placed as destination therapy; thus, we cannot assess the impact of *C auris* infection on transplant status. Although all patients died within 1 year of initial candidemia episode, 6 patients had prior LVAD-related infections along with other comorbidities, signaling an overall high-risk baseline.

In conclusion, *C auris* candidemia is a challenging infection to treat in patients with LVADs. To address this, strategies need to be developed surrounding early detection of candidemia, LVAD-specific infection prevention protocols, and source control. Early combination treatment or newer antifungal agents may be an option to clear candidemia quickly and should be evaluated with further studies.

## Notes

***Patient consent statement*.** This study does not include factors necessitating patient consent and the retrospective study design was approved by MedStar Health institutional review board.

## References

[1] Jorde UP, Saeed O, Koehl D, et al The Society of Thoracic Surgeons Intermacs 2023 annual report: focus on magnetically levitated devices. Ann Thorac Surg 2024; 117:33–44. 37944655 10.1016/j.athoracsur.2023.11.004

[2] Molina EJ, Shah P, Kiernan MS, et al The Society of Thoracic Surgeons Intermacs 2020 annual report. Ann Thorac Surg 2021; 111:778–92. 33465365 10.1016/j.athoracsur.2020.12.038

[3] Hannan MM, Husain S, Mattner F, et al Working formulation for the standardization of definitions of infections in patients using ventricular assist devices. J Hear Lung Transplant 2011; 30:375–84. 10.1016/j.healun.2011.01.717 21419995

[4] Toda K, Yonemoto Y, Fujita T, et al Risk analysis of bloodstream infection during long-term left ventricular assist device support. Ann Thorac Surg 2012; 94:1387–93. 22571882 10.1016/j.athoracsur.2012.03.021

[5] Angleitner P, Matic A, Kaider A, et al Blood stream infection and outcomes in recipients of a left ventricular assist device. Eur J Cardiothorac Surg 2020; 58:907–14. 32510152 10.1093/ejcts/ezaa153

[6] Aslam S, Hernandez M, Thornby J, Zeluff B, Darouiche RO. Risk factors and outcomes of fungal ventricular-assist device infections. Clin Infect Dis 2010; 50:664–71. 20113174 10.1086/650454 PMC3767975

[7] Kilburn S, Innes G, Quinn M, et al Antifungal resistance trends of *Candida auris* clinical isolates in New York and New Jersey from 2016 to 2020. Antimicrob Agents Chemother 2022; 66:e0224221. 35007140 10.1128/aac.02242-21 PMC8923207

[8] Clancy CJ, Nguyen MH. Emergence of *Candida auris*: an international call to arms. Clin Infect Dis 2017; 64:141–3. 27989986 10.1093/cid/ciw696

[9] CDC . Tracking Candida auris. Available at: https://www.cdc.gov/candida-auris/tracking-c-auris/index.html#:~:text=In%202022%20there%20were%202%2C377,auris. Accessed 23 September 2023.

[10] Lyman M, Forsberg K, Sexton DJ, et al Worsening spread of *Candida auris* in the United States, 2019 to 2021. Ann Intern Med 2023; 176:489–95. 36940442 10.7326/M22-3469 PMC11307313

[11] WHO Fungal priority pathogens list to guide research, development and public health action. Geneva: World Health Organization; 2022.

[12] Horton MV, Nett JE. *Candida auris* infection and biofilm formation: going beyond the surface. Curr Clin Microbiol Rep 2020; 7:51–6. 33178552 10.1007/s40588-020-00143-7 PMC7654955

[13] Pappas PG, Kauffman CA, Andes DR, et al Clinical practice guideline for the management of candidiasis: 2016 update by the Infectious Diseases Society of America. Clin Infect Dis 2016; 62:e1–50. 26679628 10.1093/cid/civ933 PMC4725385

[14] Lockhart SR, Etienne KA, Vallabhaneni S, et al Editor's choice: simultaneous emergence of multidrug-resistant *Candida auris* on 3 continents confirmed by whole-genome sequencing and epidemiological analyses. Clin Infect Dis 2017; 64:134. 27988485 10.1093/cid/ciw691 PMC5215215

[15] CDC . Treatment and management of *C. auris* infections and colonization. Available at: https://www.cdc.gov/candida-auris/hcp/clinical-care/index.html. Accessed 15 March 2023.

[16] Department of Health . Resurgence of Candida auris in the District of Columbia. Available at: https://dchealth.dc.gov/publication/april-7-2021-resurgence-candida-auris-district-columbia. Accessed 4 May 2024.

[17] Yost G, Coyle L, Gallagher C, Cotts W, Pappas P, Tatooles A. Outcomes following left ventricular assist device exchange: focus on the impacts of device infection. ASAIO J 2021; 67:642–9. 33074867 10.1097/MAT.0000000000001287

[18] Koval CE, Stosor V. Ventricular assist device-related infections and solid organ transplantation-guidelines from the American Society of Transplantation Infectious Diseases community of practice. Clin Transplant 2019; 33:e13552. 30924952 10.1111/ctr.13552

[19] Bauer TM, Choi JH, Luc JGY, et al Device exchange versus nonexchange modalities in left ventricular assist device-specific infections: a systematic review and meta-analysis. Artif Organs 2019; 43:448–57. 30357880 10.1111/aor.13378

[20] Bandara N, Samaranayake L. Emerging and future strategies in the management of recalcitrant *Candida auris*. Med Mycol 2022; 60:myac008. 35142862 10.1093/mmy/myac008 PMC8929677

[21] Hoenigl M, Sprute R, Egger M, et al The antifungal pipeline: fosmanogepix, ibrexafungerp, olorofim, opelconazole, and rezafungin. Drugs 2021; 81:1703–29. 34626339 10.1007/s40265-021-01611-0 PMC8501344

